# Exploration the therapeutic effects of sodium houttuyfonate combined with penicillin G on methicillin resistant *staphylococcus aureus* infected wounds

**DOI:** 10.3389/fphar.2025.1530217

**Published:** 2025-02-21

**Authors:** Yujie Li, Shunjie You, Sheng Li, Si Li, Aimin Jia, Xiaoyan Xiang, Shi Shen, Yan Cai

**Affiliations:** ^1^ Genetic and Prenatal Diagnosis Center, Affiliated Hospital of North Sichuan Medical College, Nanchong, China; ^2^ Department of Orthopedics, The Leshan People’s Hospital, Leshan, China; ^3^ Burn and Plastic Surgery Department of Ziyang Central Hospital, Ziyang, China; ^4^ Affiliated Hospital, North Sichuan Medical College, Nanchong, China; ^5^ Department of Orthopedics, The Affiliated Hospital, Southwest Medical University, Luzhou, China

**Keywords:** MRSA, sodium houttuyfonate, penicillin G, network pharmacology, wound healing

## Abstract

**Background:**

Methicillin resistant *Staphylococcus aureus* (MRSA) is a Gram-positive bacterium that can cause local or systemic infectious diseases, and treatment for MRSA has become a major global health issue. Sodium houttuyfonate (SH) is a natural extract of Houttuynia cordata, which has antibacterial and anti-inflammatory effects. The aim of this study is to evaluate the antibacterial effect of SH combined with penicillin G (PNC) against MRSA and its potential beneficial effects in a rat model of MRSA wound infection, and to investigate its possible mechanism in combination with network pharmacology.

**Methods:**

Evaluating the antibacterial effect of drugs through *in vitro* antibacterial experiments. Construct a MRSA infected wound model in SD rats and determine the optimal drug ratio based on the degree of wound healing. Hematoxylin & Eosin (H&E) staining was used to determine inflammatory cell infiltration, Masson staining was used to observe collagen fiber proliferation in the wound, Elisa method was used to detect inflammatory cytokine content in retrobulbar venous blood, and network pharmacology methods were used to elucidate possible molecular mechanisms.

**Result:**

The MIC of SH and PNC are 60–80 μg/mL and 40–70 U/mL, respectively. The FICI of the combined group is 0.375–0.5625, and the optimal drug ratio is SH20 μg/mL + PNC15u/mL. The number of central granulocytes infiltrated in the combined group was less than that in other groups, and the levels of IL-6 and TNF-α were significantly reduced. In addition, the collagen fibers in the wound were significantly increased. Thirteen target genes were predicted through network pharmacology, among which the core targets were IL1B, IL6, MMP9, IFNG, and TNF. SH and PNG have shown good binding potential with various targets in molecular docking. Among the 83 potential pathways of action, IL-17 signaling pathway is considered a key pathway for promoting wound healing.

**Conclusion:**

Sodium houttuyfonate combined with penicillin G can inhibit bacterial growth in MRSA infected wounds in rats, reduce neutrophil infiltration, promote collagen fiber generation in wounds, and decrease the expression levels of inflammatory factors IL-6 and TNF-α in the blood of rats after MRSA infection. It promotes wound healing through multiple targets and pathways, and preliminarily reveals the drug’s targets and molecular mechanisms.

## 1 Introduction

*Staphylococcus aureus*, which can encode penicillin binding protein 2a (PBP2a) and cannot be inactivated by β-lactam antibiotics, is called methicillin-resistant *Staphylococcus aureus* (MRSA) (Peacock and Paterson, 2015). Since its first discovery in the United Kingdom in 1961, MRSA has spread to various parts of the world and has become one of the leading pathogens causing community-acquired and healthcare related infections (Gurieva et al., 2012), it has a fast transmission speed, strong pathogenicity, and exhibits multiple drug resistance with high mortality rate (Oliveira et al., 2018). MRSA is resistant to a variety of antibiotics, such as β-lactams, fluoroquinolones, macrolides, aminoglycosides, tetracyclines, rifampicin and fusidic acid. Its drug resistance mechanism is complex, mainly including the production of β-lactamase (Lee and Park, 2016) and PBP2a (Miyachiro et al., 2019), the reduction of the permeability of antibiotics to cell membranes, the change of drug targets, the activation of drug efflux in bacteria, the inhibition of enzyme expression, the transfer of drug-resistant plasmids and the formation of bacterial biofilms (Roberts et al., 2012). Glycopeptide antibiotics are currently the “last line of defense” for the treatment of MRSA infection, but there have been intermediates between vancomycin resistant *Staphylococcus aureus* (visa) and vancomycin resistant *Staphylococcus aureus* (VRSA) (Spagnolo et al., 2014), At present, MRSA has become a major problem in clinical anti infection treatment, which requires further research on the resistance mechanism of MRSA and the development of new antibacterial treatment strategies.

The development of new antibacterial drugs faces two major challenges, namely, high costs and lengthy clinical validation time. This means that not only does it require massive financial support, but it also takes a long time to ensure the safety and effectiveness of new drugs, and the combination of multiple antibiotics may also lead to high bacterial resistance. In recent years, the therapeutic effects of traditional Chinese medicine and its active ingredients have been increasingly recognized. Traditional Chinese medicine has minimal toxic side effects, and its combination with antibiotics can reduce drug toxicity and effective dosage, reduce bacterial resistance, and provide a new approach for the clinical treatment of MRSA. Houttuynia cordata, a traditional Chinese medicine, has good anti-inflammatory activity, and is often used in clinical treatment of respiratory infections, urinary tract inflammation, acute and chronic rhinitis, conjunctivitis and other diseases (Liu et al., 2021),Its extract sodium houttuyfonate has antibacterial, antiviral, antiallergic, immune enhancing, tumor proliferation inhibiting and other effects (Zhuang et al., 2022),Numerous studies have shown that SH has a clear therapeutic effect on bacterial infections such as *Staphylococcus aureus*, *Pseudomonas aeruginosa*, *Haemophilus* influenzae, and *Streptococcus* pneumoniae (Chen et al., 2014; Wang et al., 2019), and studies have proved that a certain concentration of SH can inhibit the formation of biofilm of *Staphylococcus aureus* (Liu et al., 2011). However, its mechanism of action is not fully understood and further research is needed to elucidate its potential mechanism of action in combating infections.

Network pharmacology is an important method for exploring the potential target and molecular mechanisms of drugs, and is an emerging field of pharmacological research that integrates traditional pharmacology, bioinformatics, cheminformatics, and network biology (Liu et al., 2024; Zhu et al., 2021). By constructing a drug-disease interaction network to evaluate the molecular mechanism of drugs, we can further study the biological effects of various small molecules (Zhou et al., 2022a; Shang et al., 2023). Molecular docking technology can deeply analyze the interaction between molecules, and intuitively explain the mechanism of action between receptors and ligands in 3D graphics. Therefore, combining molecular simulation validation with network pharmacology analysis is an effective method for studying the mechanism of drug action in drug research (Wang et al., 2024).

**FIGURE 1:**
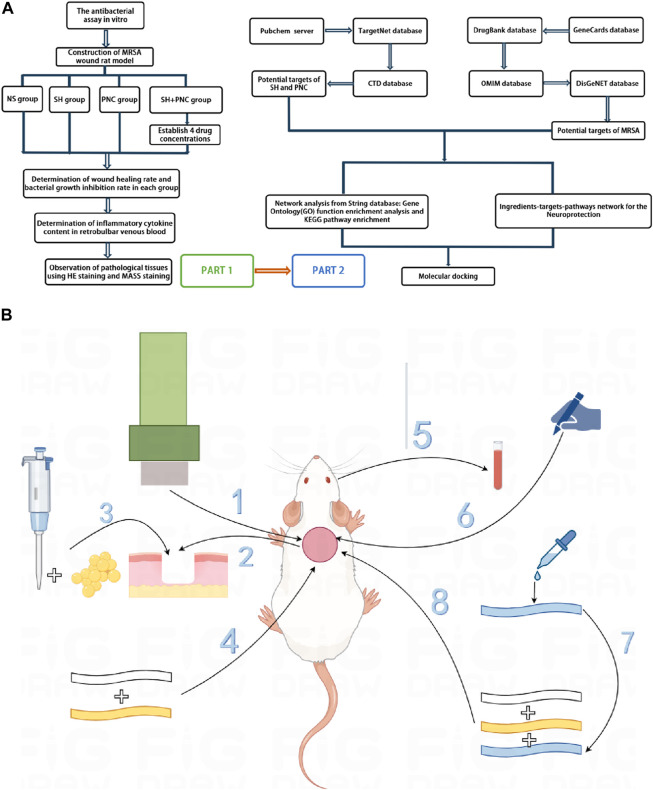
Overall workflow of SH + PNC treatment for methicillin resistant *staphylococcus aureus* infected wounds (**(A)**, Part 1 is workflow of network pharmacology; Part 2 is workflow of animal experiments). Schematic diagram of the flow of animal experiments (**(B)**, 1. Using a skin picker to create a wound on the back of the rat; 2. The wound is transverse and deep to the fascia layer.; 3. Inoculating the bacterial solution onto the back wound; 4. Wraping with vaseline gauze and sterile gauze in turn; 5. Collecting the blood from the retrobulbar vein of the rat; 6. Using a sterile transparent film to outline the wound surface; 7. Preparing drug gauze by impregnating it with drug solution; 8. Apply drug gauze, vaseline gauze, and sterile gauze to bind up the wound in order).

This study aims to investigate the antibacterial effect of sodium houttuyfonate combined with the clinically common antibiotic penicillin G, and observe its efficacy in a rat MRSA wound model. At the same time, network pharmacology and molecular docking techniques are used to predict the potential targets and action pathways of the drug, providing experimental evidence for SH to become a potential drug or antibacterial sensitizer for anti-MRSA infection in the future. The general workflow is shown in Figure 1A.

## 2 Materials and methods

### 2.1 Experimental strains and animals

Collect 20 strains of MRSA isolated from the microbiology laboratory of the Affiliated Hospital of North Sichuan Medical College from September 2020 to November 2020, with specimen types of sputum and pus. The strain was identified by VITEK GP identification card and VITEK AST-GP67 drug susceptibility card of French bioMerieux company. The quality control strain is *Staphylococcus aureus* (ATCC 25923) provided by the Microbiology Laboratory of the Affiliated Hospital of North Sichuan Medical College. The experimental animals were 55 female SD rats weighing 150–200 g each, provided by the Animal Experimental Center of North Sichuan Medical College. The ethics of animal experiments were approved by the Ethics Committee of North Sichuan Medical College (NSMC2023071).

### 2.2 Experimental drugs

Sodium houttuyfonate (purity≥99%) (Xi’an Kailai Biological Engineering Co., Ltd.); Penicillin G for injection (Shandong Lukang Pharmaceutical Co., Ltd.); Tetrazolium salt (MTT, Guangzhou Saiguo Biotechnology Co., Ltd.); dimethyl sulfoxide (DMSO, Chengdu Kelong Chemicals Co., Ltd.).

### 2.3 Strain identification

#### 2.3.1 Preparation of strain plate and bacterial solution

Prepare MH broth medium and MH agar medium and store them in a refrigerator at 4°C for future use. Thaw the standard strain of *Staphylococcus aureus* ATCC 25923 and a MRSA strain. Pipette 2 μL of bacterial solution into 1 mL of MH broth culture medium, place it in a constant temperature shaker at 37°C and 120r/min for 12 h of shaking culture. Use a loop to dip a small amount of bacterial solution and inoculate it onto the MH agar plate using a continuous serpentine curve streaking method. After 24 h of cultivation, freeze it and pick a single colony from the plate with the inoculated strain into 1 mL of MH broth culture medium. Centrifuge the cultured bacterial solution at 8000r/min for 3 min, and then add 1 mL of normal saline to resuspend it. Use physiological saline as a blank control, detect the OD600 nm value of the bacterial solution using a nucleic acid protein analyzer, dilute the OD value of the bacterial solution with physiological saline to 1.0 (1OD600 = 1.5 × 108 cfu/mL), and finally dilute the bacterial solution with MH broth medium to 1 × 105 cfu/mL for use.

#### 2.3.2 MRSA identification

Adjust the concentration of the bacterial solution to 0.5 McFarland concentration using a turbidimeter, dip a sterile cotton swab into the bacterial suspension, then evenly spread it onto the MH agar plate, and finally attach a cefoxitin (FOX) drug susceptibility disk (30 μg/disk). Incubate for 24 h in a 37°C incubator. The results were interpreted in accordance with the Clinical and Laboratory Standards Institute (CLSI) standards. The quality control strain is *Staphylococcus aureus* ATCC 25923.

### 2.4 *In vitro* antibacterial experiment

#### 2.4.1 Drug preparation

Add sodium houttuyfonate powder and penicillin G powder to the MH broth culture solution, shake and mix well, and prepare according to the two-fold dilution method. The concentration gradients are 1,600, 800, 400, 200, 100, 50, 25, 12.5 μg/mL and 1,200, 600, 300, 150, 75, 37.5, 18.75, 9.375 μg/mL. Weigh 250 mg of MTT powder and dissolve it in 50 mL of PBS solution that has been sterilized under high pressure. Prepare a MTT solution with a concentration of 5 mg/mL, filter it through a 0.25 μm bacterial filter, and store it in a refrigerator at 4°C away from light.

#### 2.4.2 Determination of minimum inhibitory concentration (MIC)

Take a sterile 96-well plates, set up 3 replicates for each drug concentration, and do not add samples to the edge wells of the 96-well plates. Add the diluted drugs in the order from high concentration to low concentration, except for the negative control, and add bacterial solution (concentration of 1 × 105 cfu/mL) to each well. Use MH broth culture solution to make up to 100ul, and the final concentrations of SH solution are 640, 320, 160, 80, 40, 20, 10, 5 μg/mL, and PNC is 480, 240, 120, 60, 30, 15, 7.5, 3.75u/mL. The positive control group consists of MRSA bacteria without drug intervention, while the negative control group consists of pure MH broth medium. The remaining unsampled edge holes were filled with physiological saline. Place it in a 37°C incubator for 15 h, then add 10 μL of MTT solution to each well and shake it for 1 h at 37°C on a shaker (120r/min). The minimum drug concentration corresponding to the absence of bacterial growth in the visual inspection is the MIC of the bacteria. Finally, add DMSO to each well, and use a microplate reader to measure the absorbance value at a wavelength of 490 nm, thereby calculating the inhibition rate of each concentration of the two drugs.

#### 2.4.3 Combined antibacterial effect of drugs

According to the above MIC results, the final concentrations of the two drugs after sample addition were 2MIC, 1MIC, 1/2MIC, 1/4MIC, 1/8MIC, 1/16MIC, and 1/32MIC. Using the chessboard dilution method, add 40ul of each drug and 20ul of bacterial solution to each well, and make up to 100ul with MH broth culture medium. Add 100ul of normal saline to the hole without sample on the edge。Use the same method as above and calculate the MIC and fractional inhibitory concentration index (FICI). Standard: FICI ≤ 0.5 indicates synergistic effect; 0.5 < FICI ≤ 1 is additive effect; 1 < FICI ≤ 2 is an unrelated effect; FICI > 2 is antagonistic.

### 2.5 Animal experiments

#### 2.5.1 Establishment of rat model of MRSA infection wound

SD rats were injected with 3% sodium pentobarbital solution intraperitoneally at a dose of 0.3 mL/100 g. After complete anesthesia, shave the back hair, with an area of 3 × 3 cm^2^. Create a circular wound with a diameter of 15 mm using a skin punch, reaching the fascia. Then, use a pipette to inject 15 μL of bacterial solution with a concentration of 1.5 × 10^8 cfu/mL into the back wound of an SD rat. Once the bacterial solution is fully absorbed, sequentially apply a single layer of sterile vaseline gauze for moisturizing and cover it with four layers of sterile gauze (Figure 1B). Breding in the animal experimental center of Southwest Medical University, with the ambient temperature of 22°C–24°C and the relative humidity of 60%–80% manually controlled.

#### 2.5.2 Identification of wound secretion

On the third day after surgery, the wound secretion was taken with a cotton swab and transferred to a methicillin-resistant *Staphylococcus aureus* color medium, which was evenly applied and incubated for 24 h in a 37°C incubator. The results were identified according to the instructions of the colorimetric medium for methicillin-resistant *Staphylococcus aureus*.

#### 2.5.3 Animal grouping and preparation of drug gauze

Prepare the drug solution according to the results of the *in vitro* antibacterial test, take a sterile double-layer gauze measuring 2 × 2 cm^2^, and soak it with the drug solution. SD rats were divided into five groups, with three rats in each group. Four of the groups were treated with different concentrations of SH + PNC, while one group was treated with normal saline as a blank control group.

#### 2.5.4 Measurement of wound healing rate

The rats were subjected to dressing changes on postoperative days 3, 5, and 7, and were sacrificed on day 9. On the 3rd and 9th day, sterile transparent film was placed on the wound surface, and then the wound surface was outlined and scanned using a gel imaging system. The image was analyzed using ImageJ software to calculate the wound area and wound healing rate. The wound healing rate (%) = (area before treatment - area after treatment)/area before treatment X100%.

#### 2.5.5 HE staining and MASSON staining

On the ninth day, SD rats were sacrificed and skin tissue was removed using a 15 mm diameter skin-removing device aligned with the center of the wound, which reached deep into the fascia. Soak the skin tissue in the fixative for 24 h, and embed the tissue in paraffin. Then, the tissue was stained with HE and MASSON, and the number of neutrophils in a random field of view was observed and recorded under a 200x light microscope. The percentage of collagen fibers in the image was determined using ImageJ software.

#### 2.5.6 Inflammatory factor determination

On the 3rd and 9th day, anesthetize rats with 3% sodium pentobarbital intraperitoneally, insert the capillary glass tube from the inner canthus, slide it to the retrobulbar vein, rotate the glass tube until blood drips out, collect 1 mL, then pull out the glass tube, place the whole blood sample at room temperature for 2 h, take the supernatant, freeze it and store it in a −80°C refrigerator for future use. Use the corresponding ELISA kit instructions, and finally measure the optical density (OD450 nm) value and calculate the sample concentration.

### 2.6 Network pharmacology

#### 2.6.1 Target acquisition

The chemical structures of SH and PNC were obtained on Pubchem database (https://pubchem.ncbi.nlm.nih.gov/) (Kim, 2016). Upload SH and PNC to the TargetNet database (http://targetnet.scbdd.com/) (Zhi-Jiang et al., 2016) and CTD database (Comparative Toxicogenomics Database, https://ctdbase.org/) (Tumayhi et al., 2023) to obtain the target sites of action, load the obtained targets into the UniProtKB database (https://www.uniprot.org/) (Zaru et al., 2020) to obtain standard target names, and set the search criteria to “*Homo Sapiens*”. Search for ‘methicillin-resistant *Staphylococcus aureus* infection’ in the GeneCards database (https://www.genecards.org/) (Stelzer et al., 2016), Drug Bank database (https://go.drugbank.com/) (Svensson et al., 2018), DisGeNET database (https://disgenet.com/) (Piñero et al., 2017), and OMIM database (https://www.omim.org/) (Amberger et al., 2019) to obtain disease-related genes. Integrate data, remove duplicate values, and cross-reference the above targets using R software (Version 4.4.1, The R Project for Statistical Computing) (Sepulveda, 2020; Bota and Fodor, 2019) to obtain potential therapeutic targets.

#### 2.6.2 Network construction

Import the cross targets into the STRING database (Version 12.0, https://cn.string-db.org/) (Szklarczyk et al., 2023) to construct a protein–protein interaction (PPI) network, with the condition set to *Homo sapiens*, minimum required high confidence (0.700) and hide disconnected nodes in the network. Further analysis of the PPI network was conducted through the Cytoscape software (Version 3.10.2) (Doncheva et al., 2019) to identify core targets.

#### 2.6.3 Functional enrichment analyses and compound-target-pathways (CTP) network

Go analysis and KEGG pathway analysis of SH and PNC targets were performed by using DAVID database (https://david.ncifcrf.gov/home.jsp) (Xie et al., 2022) to predict the biological functions, action pathways and potential relationships of SH and PNC. Based on these core targets and pathways, CTP network were constructed to intuitively clarify the therapeutic mechanism of SH + PNC on methicillin resistant *staphylococcus aureus* infected wounds.

#### 2.6.4 Molecular docking

Paymol software (Version 3.0) (Hancock et al., 2022) was used to remove water molecules and small molecule ligands from the protein structure, and then imported into AutoDock Vina (Version 1.1.2) (Forli et al., 2016) for hydrotreating. The receptor and ligand were docked in AutoDock Vina software to analyze their binding activities. Paymol was used to visualize the results of molecular docking simulation validation.

### 2.7 Statistical methods

SPSS27.0 statistical software was used for analysis. Data were expressed as $\bar{x}\pm s$ , and data that conformed to normal distribution and homogeneity of variance were analyzed using one-way analysis of variance and independent sample t-test. LSD-t test was used for inter-group comparison Sample data that do not conform to the normal distribution are represented by the median (interquartile range), and Kruskal–Wallis H tests are used for overall comparisons and pairwise comparisons between sample groups. P < 0.05 indicates a statistically significant difference.

## 3 Result

### 3.1 Identification of MRSA strains

**FIGURE 2:**
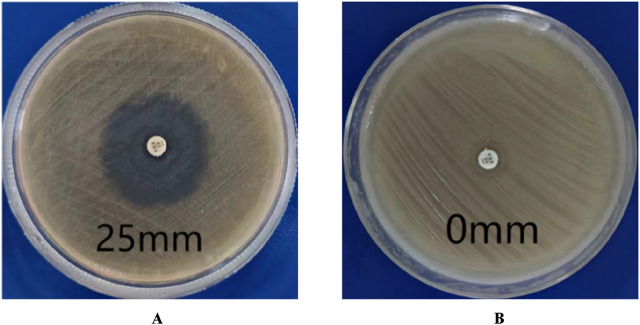
Drug sensitivity test results. **(A)** is MSSA (the diameters of bacterial inhibition zone≥22 mm). **(B)** is MRSA (the diameters of bacterial inhibition zone <21 mm).

According to CLSI standards, when the diameter of the inhibition zone is ≤ 21 mm, it can be determined as MRSA; When the diameter of the bacteriostatic zone is ≥ 22 mm, it can be diagnosed as methicillin-sensitive *Staphylococcus aureus* (MSSA). The inhibition zone test was performed on the quality control strain ATCC 25923, and the inhibition zone diameter was 25 mm (Figure 2A), which meets the MSSA standard. The diameter of the inhibition zone for the clinical strains was less than 21 mm (Figure 2B), thus confirming them as MRSA.

### 3.2 Antibacterial testing *in vitro*

**FIGURE 3:**
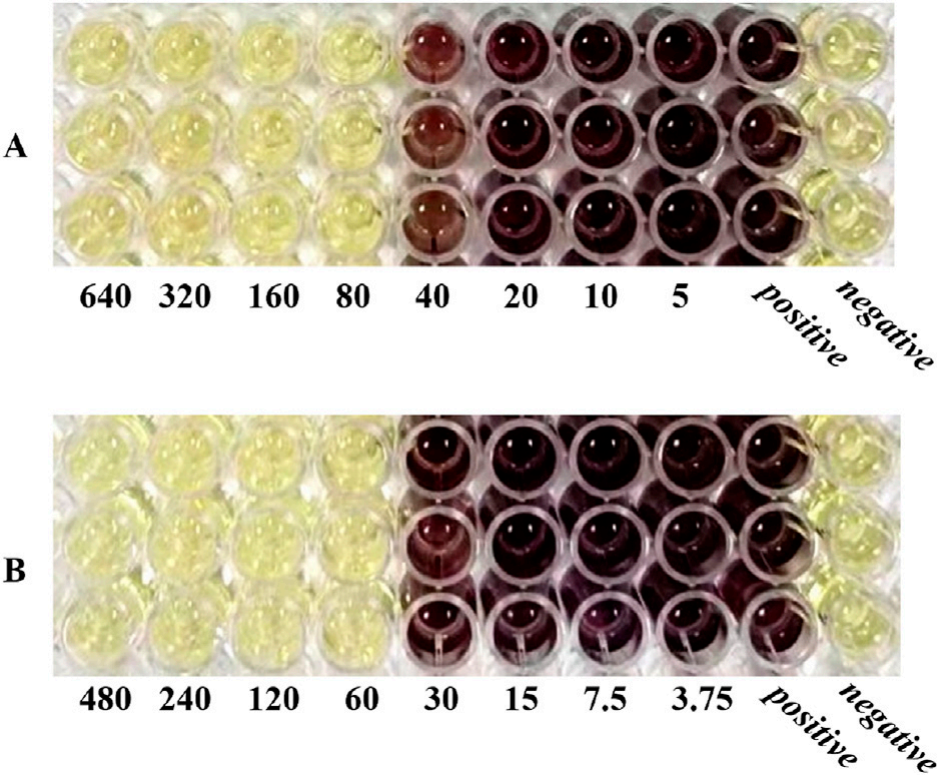
Microbroth dilution method was used to determine the MIC of drugs. **(A)** is SH(μg/mL). **(B)** is PNC(u/mL).

**FIGURE 4:**
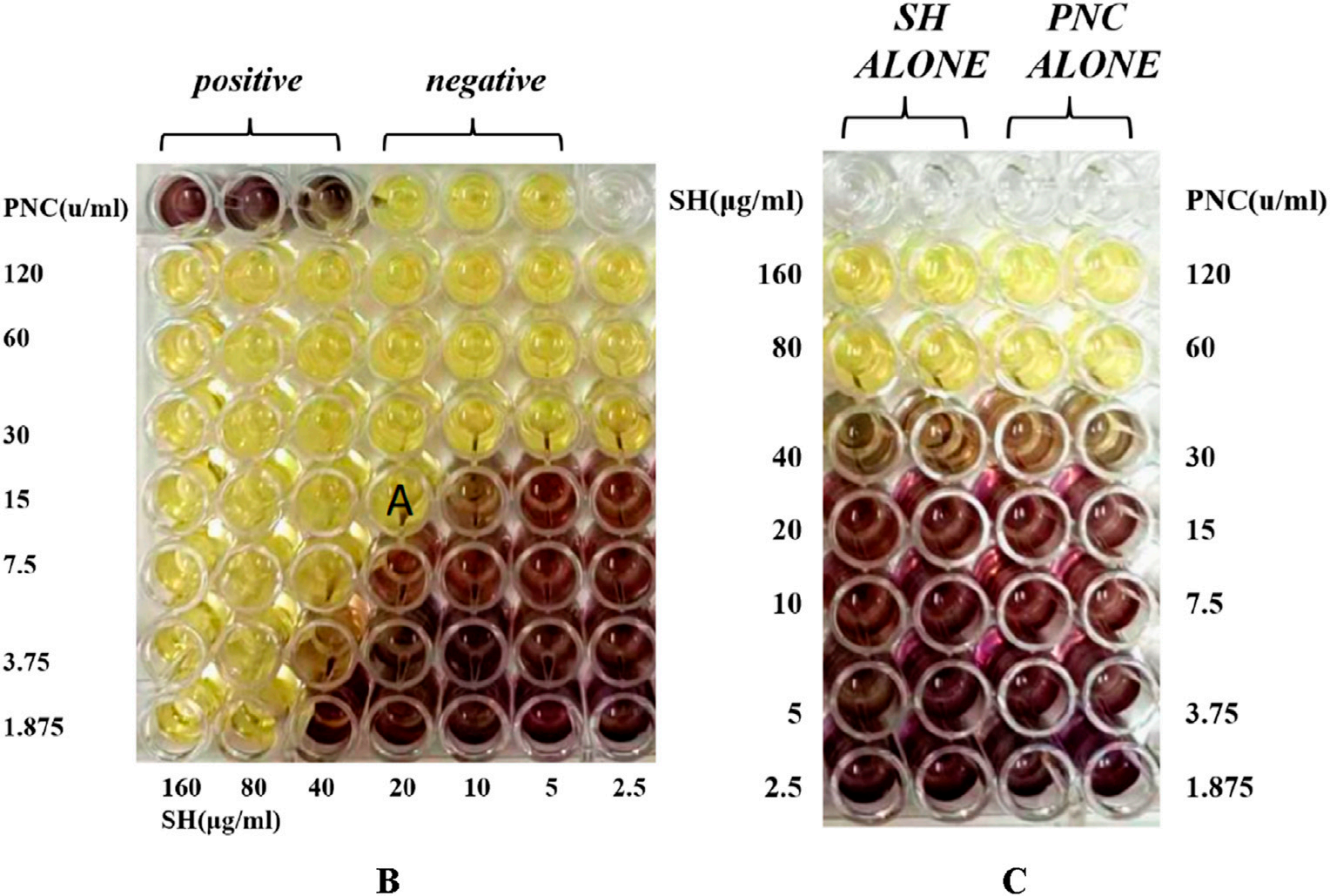
Drug sensitivity results of Sodium houttuyfonate combined with Penicilin G detected by microbroth dilution method (**(B)**, Combined drug sensitivity results of strain No. 1). The first row of 1-3 holes are the positive control group, and the 4-6 holes are the negative control group. The MIC of SH alone in the figure is 80 μg/mL, and the MIC of PNC alone is 60u/mL. The best bacteriostatic concentration combination (SH 20 μg/mL + PNC15u/mL) is shown in the hole **(A)** in the figure where no bacterial growth is observed, with FICI = 20/80 + 15/60 = 0.5. Determination of MIC values for SH and PNC of isolated strain No. 1 **(C)**.

**FIGURE 5:**
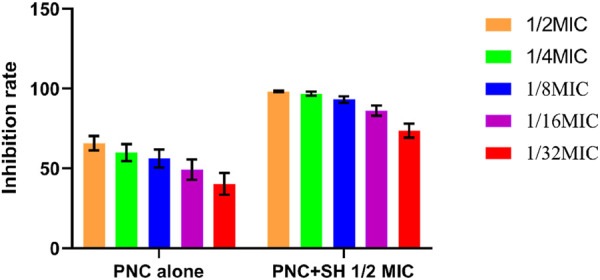
Changes in average inhibition rate of Penicilin G alone and combination with Sodium houttuyfonate (1/2 MIC).

The MIC values of SH and PNC against the standard strain of *Staphylococcus aureus* ATCC 25923 were 60 μg/mL and <1u/mL, respectively. The MIC values for clinical isolates of MRSA ranged from 60 to 80 μg/mL and 40 to 70u/mL (Figure 3, Table 1). The FICI of SH + PNC was 0.375–0.5625, and 14 strains showed synergistic antibacterial effects, while 6 strains showed additive antibacterial effects (Figure 4, Table 1). The OD value of the 96-well plates at a wavelength of 490 nm was measured using a microplate reader, and the average inhibition rate was calculated. The results showed that the inhibition rate of sodium houttuyfonate at a drug concentration of 1/2MIC combined with different concentrations of penicillin G was significantly higher than that of penicillin G alone (P < 0.05), indicating that SH can significantly increase the antibacterial effect of PNC(Figure 5, Table 2).

**TABLE 1:** MIC and FICI values of various drugs *in vitro* antibacterial experiments.

Strain number	SH alone MIC(μg/mL)	PNC alone MIC(u/mL)	SH MIC(μg/mL) /PNC MIC(u/mL)	FICI
1	80	60	20/15	0.5
2	70	60	4.375/30	0.5625
3	80	70	10/17.5	0.375
4	60	70	3.75/35	0.5625
5	80	60	10/15	0.375
6	60	60	3.75/30	0.5625
7	60	60	15/15	0.5
8	60	60	15/15	0.5
9	70	70	4.375/35	0.5625
10	70	70	17.5/17.5	0.5
11	60	60	3.75/30	0.5625
12	60	60	15/15	0.5
13	60	40	7.5/10	0.375
14	60	40	7.5/10	0.375
15	60	40	7.5/10	0.375
16	60	40	15/10	0.5
17	60	40	7.5/10	0.375
18	60	40	3.75/20	0.5625
19	60	40	15/10	0.5
20	60	40	15/10	0.5

**TABLE 2:** Comparison of average inhibition rates of Penicilin G alone and combined with Sodium houttuyfonate (1/2 MIC) ( $\bar{x}\pm s$ , %).

PNC	Inhibition rate (PNC)	Inhibition rate (PNC + SH)	t
1/2MIC	65.74 ± 4.58	98.23 ± 0.48	31.55
1/4MIC	59.89 ± 5.27	96.80 ± 1.26	30.46
1/8MIC	56.18 ± 5.63	93.16 ± 1.98	27.71
1/16MIC	49.20 ± 6.40	86.19 ± 3.21	23.10
1/32MIC	40.35 ± 6.82	73.64 ± 4.33	18.42

### 3.3 Identification of wound secretion

**FIGURE 6:**
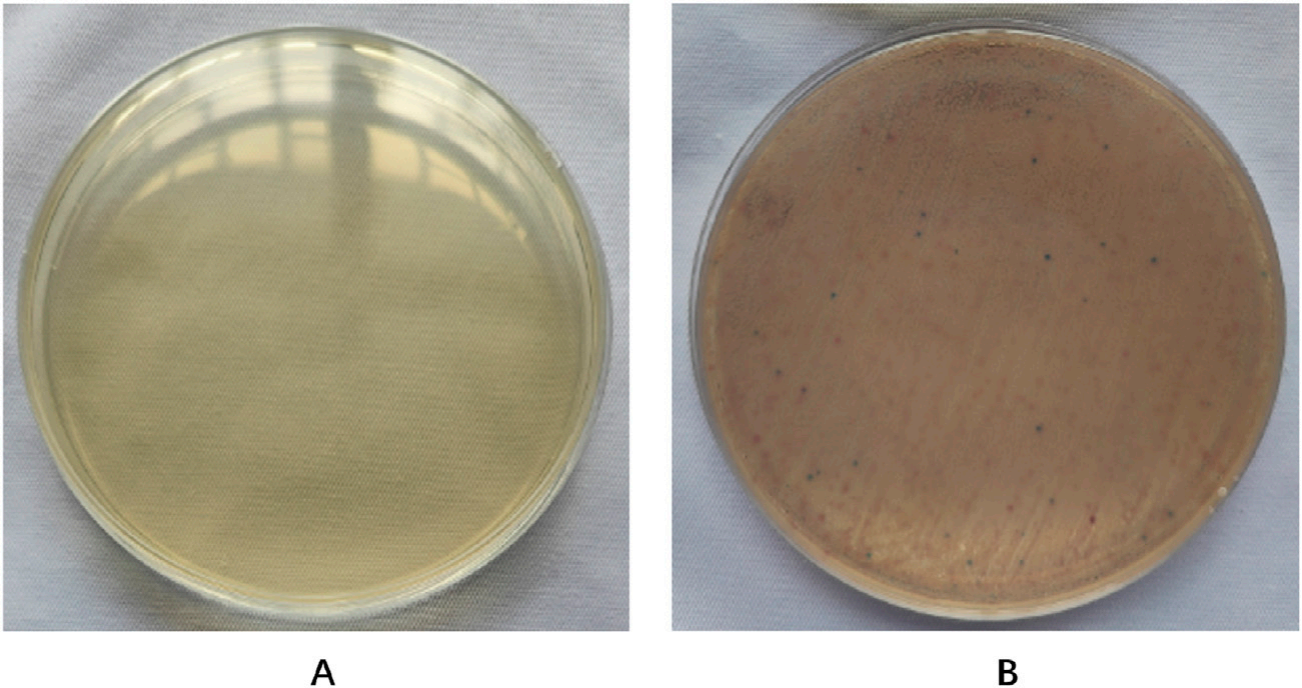
Results of MRSA chromoculture plate experiments. The color of the uninoculated MRSA coloration culture plate is light yellow **(A)**. A large number of pink MRSA colonies grow on the plate, scattered among other blue **(B)** colonies.

According to the instructions for the color medium for methicillin-resistant *Staphylococcus aureus* (MRSA) from the French company Comag, MRSA colonies are pink to light purple; Other bacterial colonies are blue. The results showed that the colonies of bacteria cultured from the wound secretions in each group were almost pink MRSA, with occasional blue colonies of other bacteria (Figure 6).

### 3.4 Drug proportioning scheme

**FIGURE 7:**
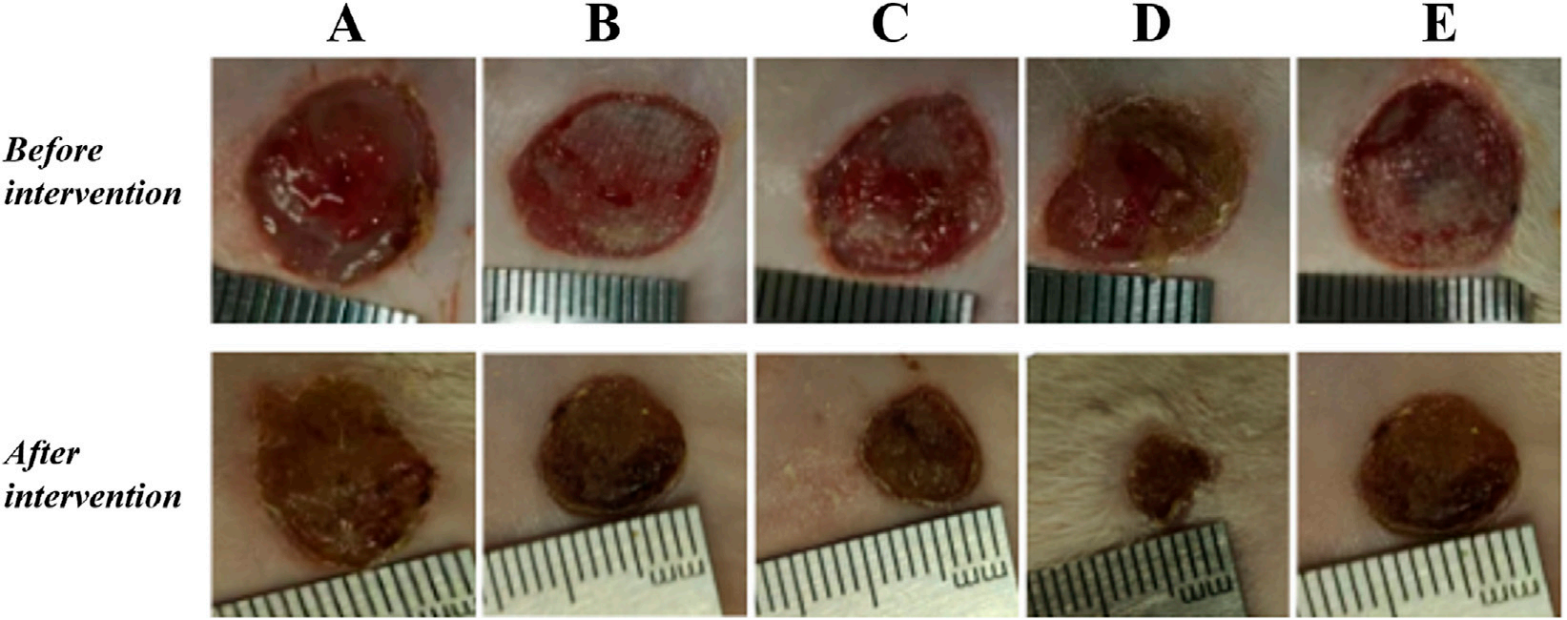
The healing status of MRSA wounds under different medication regimens of SH + PNC. **(A)** SH5 μg/mL + PNC15u/mL, **(B)** SH5 μg/mL + PNC30u/mL, **(C)** SH20 μg/mL + PNC15u/mL, **(D)** SH20 μg/mL + PNC30u/mL, **(E)** Normal saline (NS) was used as the blank control group.

The results showed that there was redness and swelling around the wound surface in all groups before intervention, accompanied by a large amount of purulent exudation, and all groups had obvious bacterial mats. After intervention, the wound surface area in experimental group C and experimental group D was smaller than that in the control group, and no secretions or bacterial mats were observed (Figure 7). The healing rate of each group is shown in Table 3. The Dunnett-t test showed that the wound healing rate in experimental groups C and D was significantly higher than that in the control group (P < 0.05) (Table 4). Group C had a concentration of 90.78 ± 8.93 (%), while the group D had a concentration of 91.98 ± 9.77 (%). There was no significant difference between the two groups, and both were significantly higher than the other three groups. At the same time, the drug concentration of penicillin G in group C was lower than that in group D, which allowed group C to reduce the amount of antibiotic usage while achieving similar wound healing rates. Therefore, the drug concentration of experimental group C (SH20 μg/mL + PNC15u/mL) was selected for subsequent experiments.

**TABLE 3:** The healing rate of MRSA wounds under different medication regimens of SH + NPC.

Group (n = 15)	Healing rate (%)	*F*	*P*
A	28.37 ± 8.63	46.64	<0.0001
B	29.67 ± 8.57		
C	90.78 ± 8.93		
D	91.98 ± 9.77		
E (NS)	28.64 ± 8.21		

**TABLE 4:** Comparison of MRSA wound healing rate within the SH + PNC therapy group.

Group (n = 15)	Healing rate (%)	*P*
A	28.37 ± 8.63	0.98
B	29.67 ± 8.57	0.96
C	90.78 ± 8.93	<0.0001
D	91.98 ± 9.77	<0.0001

### 3.5 Wound healing rate

**FIGURE 8:**
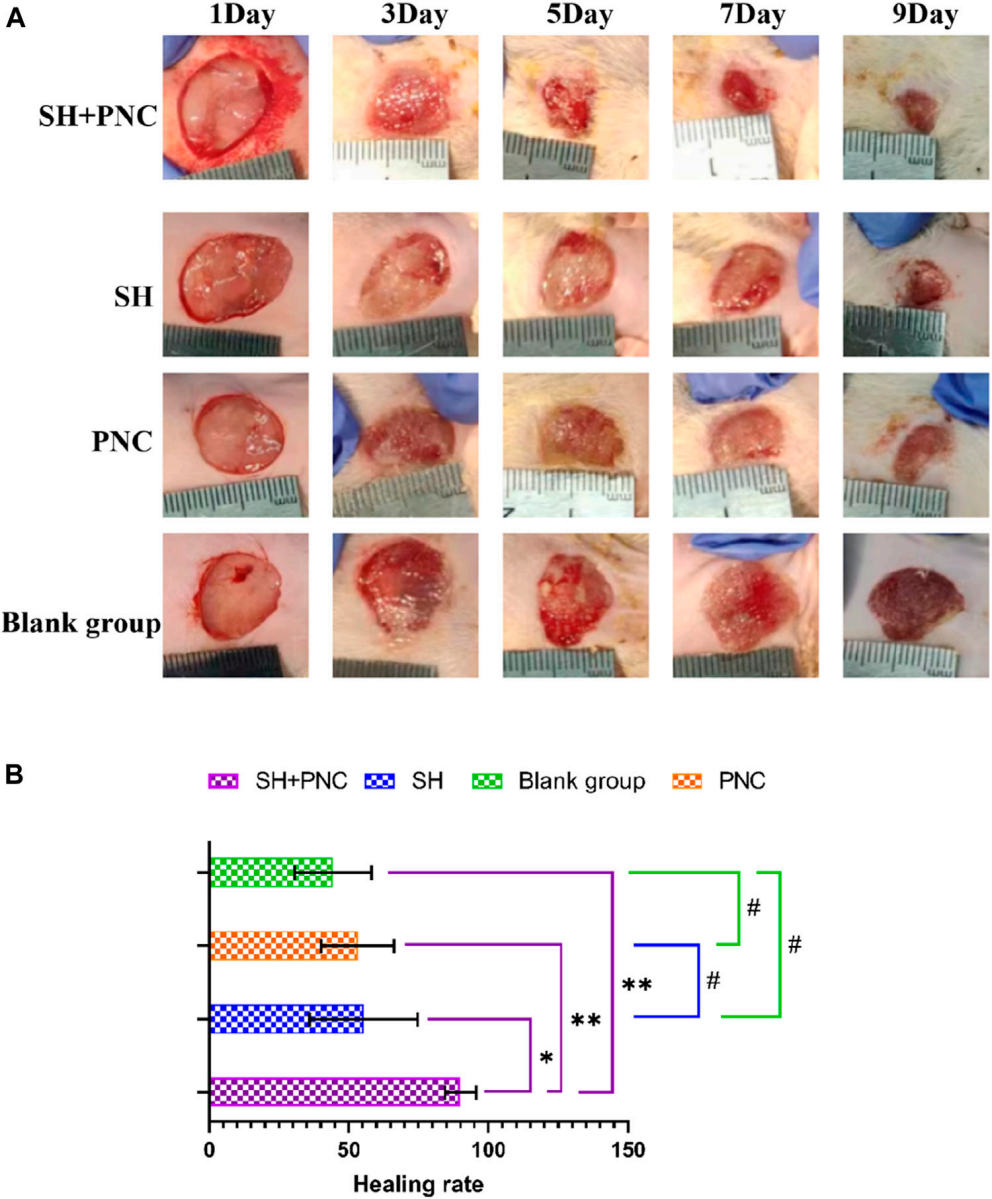
Contrast of infected wounds in MRSA (**(A)**, Photography equipment: SONY IMAX799 ISO:500 WB:6,000 Shutter:1/30 *1). Comparison of wound healing rates between the groups (**(B)**, *p < 0.05, **p < 0.01, #p > 0.05, p < 0.05 indicates statistical significance, while a p < 0.01 indicates a significant difference).

Compared with other groups, the SH + PNC group showed a significant reduction in wound area on day 5, with less purulent secretion and no bacterial mats observed. The wound area in the SH group significantly decreased on day 9. The wound area in the PNC group decreased on day 7, but there was no significant change in purulent secretion compared with the blank control group. It can be seen that the healing time of MRSA infected wounds in the combined medication group is faster and the healing effect is better (Figure 8A).

ImageJ software was used to process the images, and the wound area of each group was measured, and the wound healing rate of each group was calculated (Figure 8B, Table 5). The LSD-t test showed that the wound healing rate in the SH + PNC group was significantly higher than that in the other groups (P < 0.05), while there was no significant difference in wound healing rate between the SH, PNC, and blank groups (P > 0.05).

**TABLE 5:** Comparison of wound healing rates between the groups.

Group (n = 20)	Healing rate (%)	*F*	*P*
SH + PNC	90.08 ± 5.64	46.65	<0.001
SH	55.35 ± 19.39		
PNC	53.19 ± 13.05		
Blank group	44.38 ± 13.73		

### 3.6 Inflammatory factor assay

**FIGURE 9:**
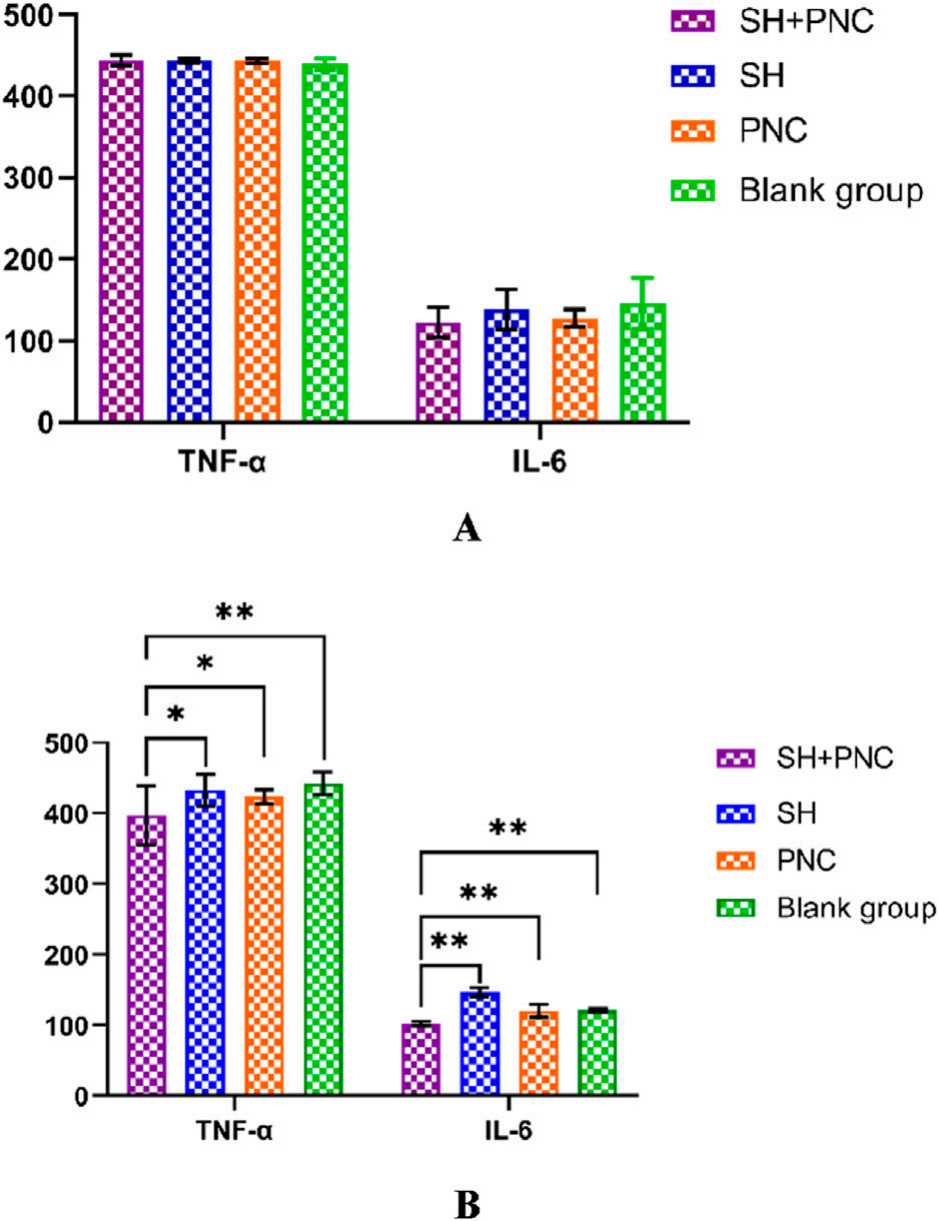
Comparison of IL-6, and TNF- α in blood between the groups before intervention **(A)**. Comparison of IL-6, and TNF- α in blood between the groups after intervention **(B)** (*p < 0.05, **p < 0.01, p < 0.05 indicates statistical significance, while a p < 0.01 indicates a significant difference).

There were no significant differences in the levels of IL-6, INOS, and TNF-α in the blood of each group before intervention, while there were significant differences in the levels of IL-6 and TNF-α in the blood of each group after intervention (P < 0.05) (Table 6; Figure 9).

**TABLE 6:** Comparison of IL-6 and TNF- α in blood between the groups.

Group (n = 40)	TNF-α (ng/L)	IL-6 (ng/L)	
SH + PNC	444.13 ± 6.69	122.80 ± 18.37	Before intervention
SH	443.83 ± 2.86	138.03 ± 24.54	
PNC	443.83 ± 3.12	127.77 ± 10.45	
Blank group	439.19 ± 7.11	145.75 ± 31.20	
F	1.99	2.1	
P	0.13	0.12	
SH + PNC	397.49 ± 41.35	102.13 ± 3.31	After intervention
SH	433.62 ± 22.52	147.39 ± 5.87	
PNC	423.71 ± 10.11	120.75 ± 8.64	
Blank group	442.82 ± 16.18	121.55 ± 2.48	
F	5.94	109.668	
P	0.012	0.009	

Using the LSD-t test, it was found that the levels of IL-6 and TNF-α in the blood of the combined medication group were significantly lower than those of the other groups after intervention (P < 0.05). The IL-6 content in the SH group was lower than that in the PNC group and the blank control group (P < 0.05). There was no significant difference in the levels of IL-6 and TNF-α in the blood between the PNC group and the control group.

### 3.7 HE staining

**FIGURE 10:**
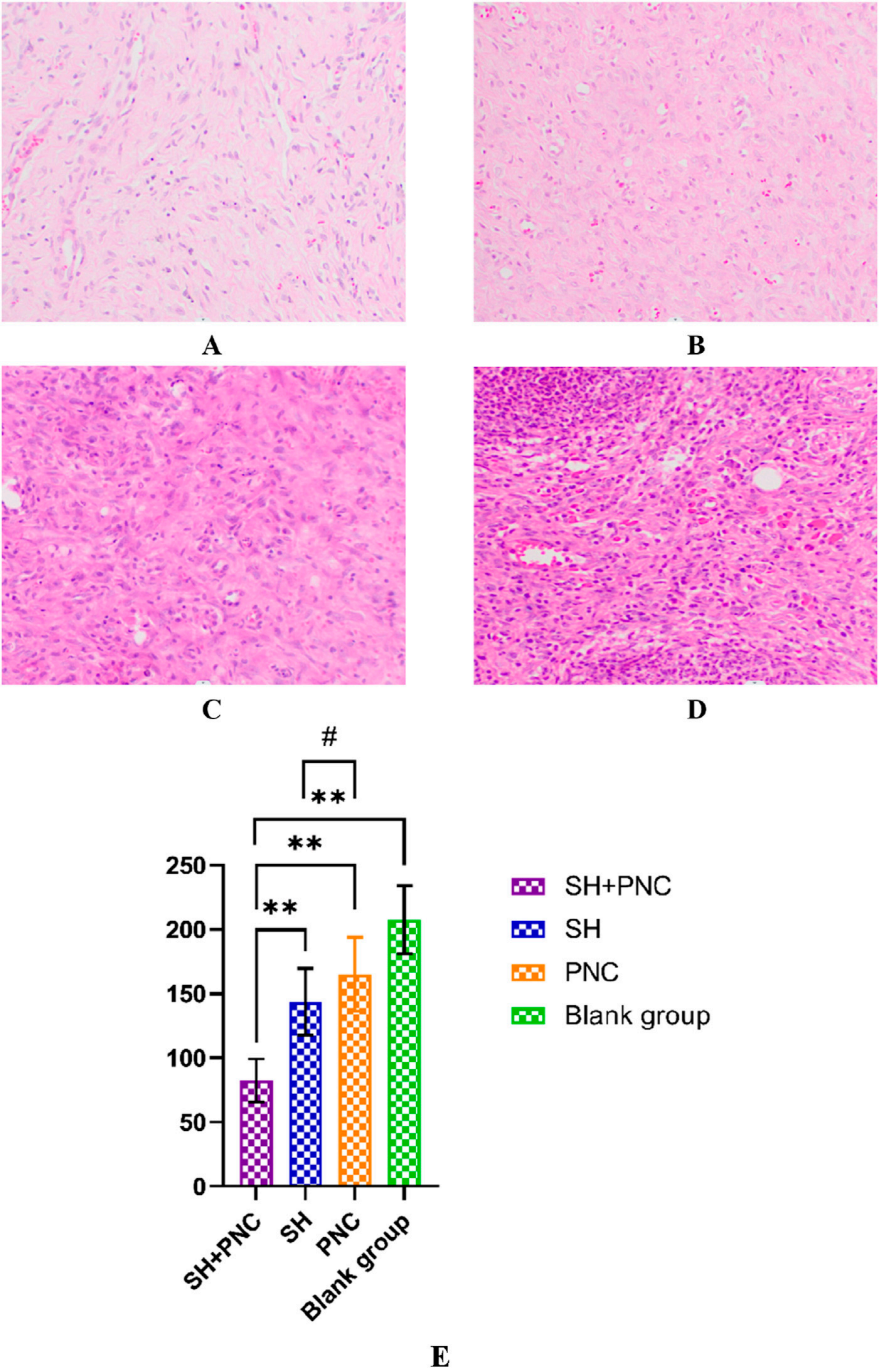
HE stained wound tissue pathology sections from MRSA. **(A)** SH + PNC, **(B)** SH, **(C)** PNC, **(D)** Blank group (x100). Comparison of neutrophil numbers in MRSA infected wounds (**(E)**, **p < 0.01, p < 0.05 indicates statistical significance, while a p < 0.01 indicates a significant difference).

Under 100x light microscope, neutrophils were clearly identified based on their microscopic characteristics (Figure 10). Record the average value of the number of neutrophils under random vision (Table 7). The LSD-t test was used to compare the groups, showing that the number of neutrophils in the combined medication group was significantly lower (P < 0.05). There was no significant difference in the number of neutrophils between the SH group and the PNC group.

**TABLE 7:** Comparison of neutrophil numbers in MRSA infected wounds.

Group (n = 40)	Piece	F	P
SH + PNC	82.40 ± 16.59	43.793	<0.001
SH	143.50 ± 25.97		
PNC	165.20 ± 28.70		
Blank group	207.50 ± 26.58		

### 3.8 Masson staining

**FIGURE 11:**
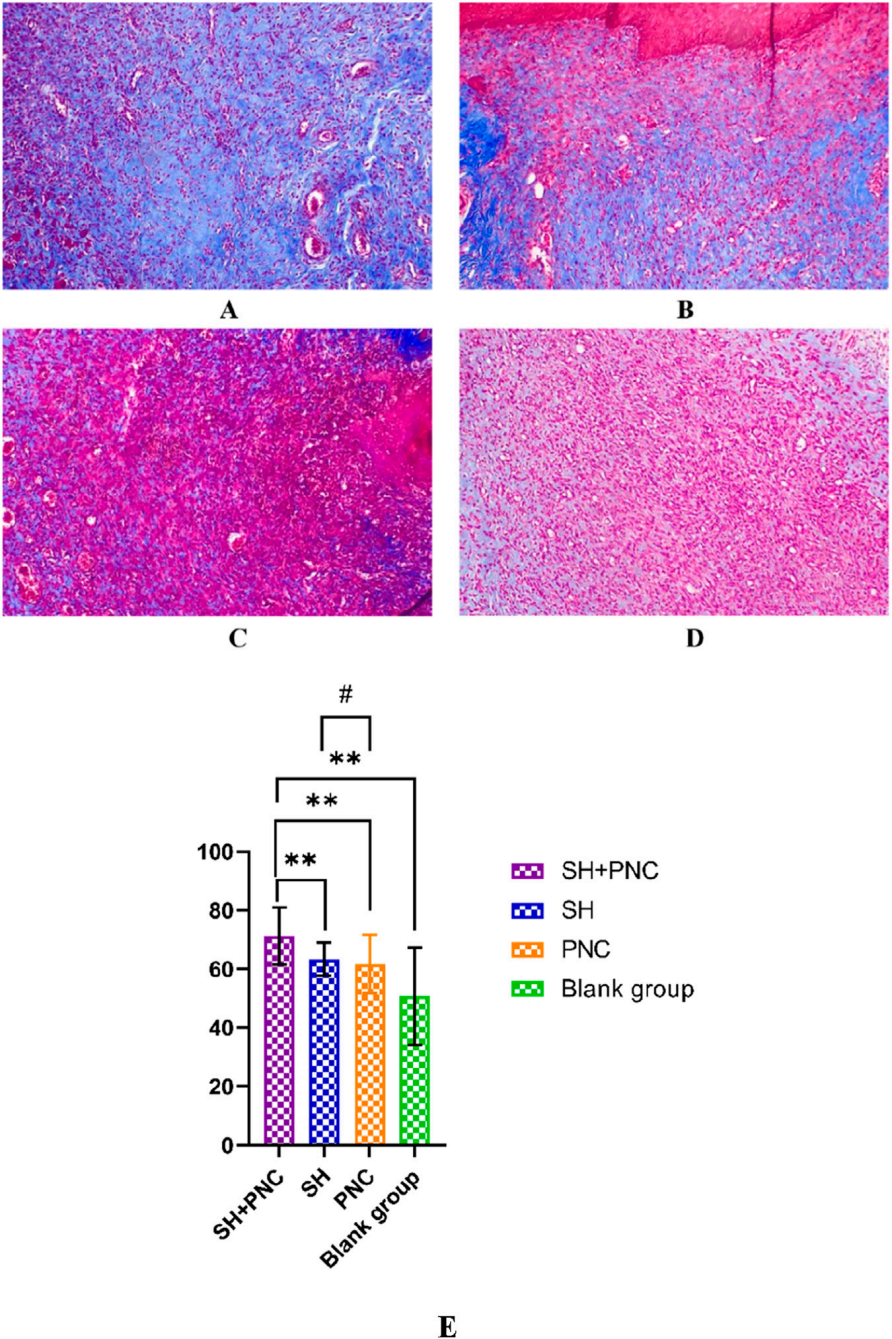
Masson-stained wound tissue pathology sections from MRSA. **(A)** SH + PNC, **(B)** SH, **(C)** PNC, **(D)** Blank group (x100). Comparison of percent collagen fibers of infected wounds in MRSA (**(E)**, **p < 0.01, p < 0.05 indicates statistical significance, while a p < 0.01 indicates a significant difference).

As shown in Figure 11, blue represents collagen fibers. Randomly record the image of the field of view, use the ImageJ software to determine the percentage of collagen fibers in the image, and take the average value (Table 8). The Kruskal–Wallis H test was used for intra-group comparison, and the percentage of collagen fibers in the combination group was the highest (P < 0.05). There was no significant difference in the percentage of collagen fibers between the SH group and the PNC group.

**TABLE 8:** Comparison of percent collagen fibers of infected wounds in MRSA.

Group (n = 40)	Percentage of collagen fibers (%)	H	P
SH + PNC	71.2 ± 9.69	23.57	<0.001
SH	63.4 ± 5.65		
PNC	61.7 ± 9.90		
Blank group	50.7 ± 16.57		

### 3.9 Network pharmacology

#### 3.9.1 The targets information of SH and PNC

Using PubChem database, we obtained the 3D structure and smiles chemical formula of SH and PNC. By searching the database separately for keywords’ sodium houttuyfonate “and” penicillin G ′, we obtained 83 and 325 relevant targets, respectively. Upload the above targets to UniProtKB database to get the target standard target names.

#### 3.9.2 Network construction

**FIGURE 12:**
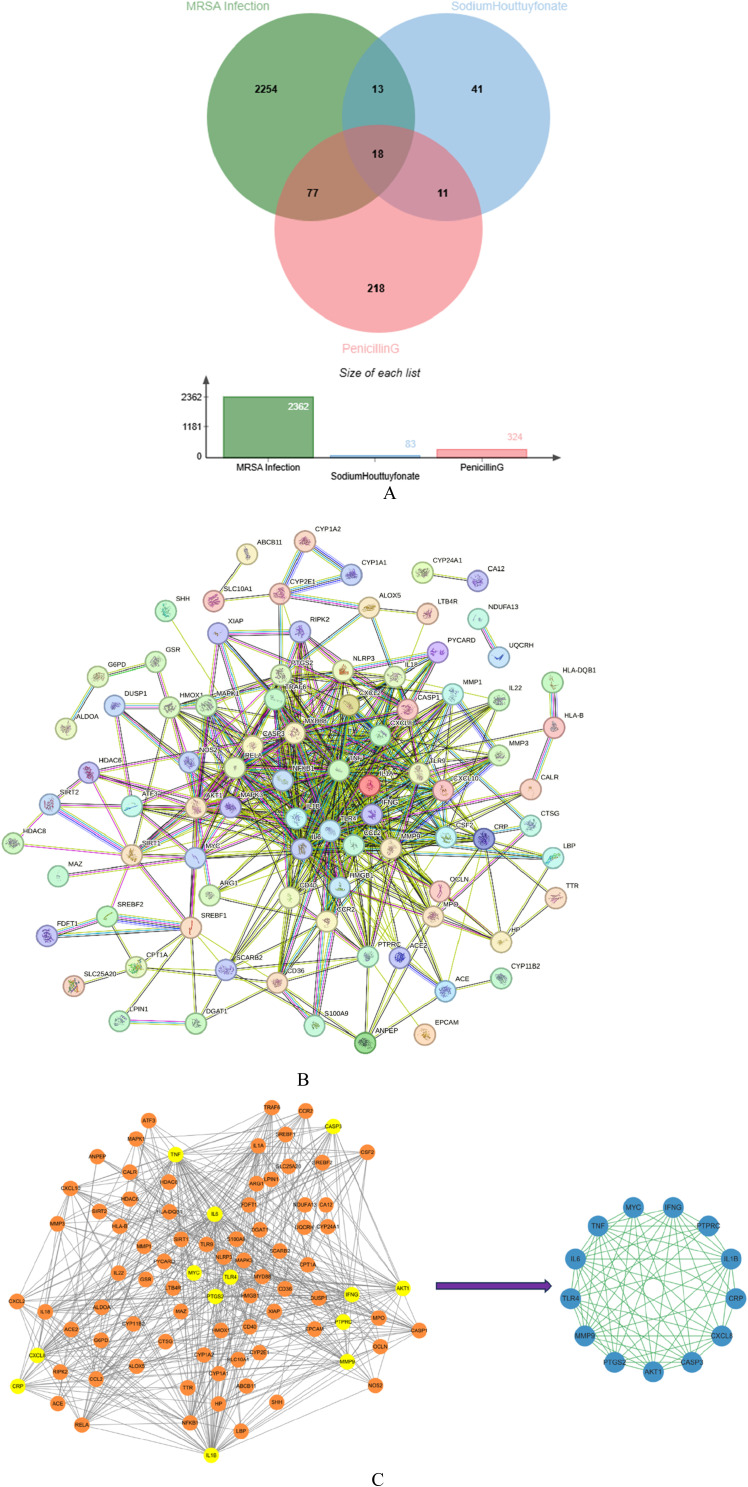
Venn diagram of Sodium houttuyfonate and penicillin G **(A)**. PPI network of potential therapeutic targets **(B)**. Import PPI network data into Cytoscape to obtain protein interaction network, the key target protein interaction network obtained after filtering by CytoNCA analysis **(C)**.

2,362 disease-related targets were obtained by using GeneCards database, Drug Bank database, OMIM database and DisGeNET database. This indirectly reflects the complex molecular pathways and related mechanisms involved in nerve injury. Using Cytoscape software, we interacted SH and PNC targets with disease targets and obtained Venn diagram (Figure 12A).

**TABLE 9:** Comparison of percent collagen fibers of infected wounds in MRSA.

Group (n = 40)	Percentage of collagen fibers (%)	H	P
SH + PNC	71.2 ± 9.69	23.57	<0.001
SH	63.4 ± 5.65		
PNC	61.7 ± 9.90		
Blank group	50.7 ± 16.57		

We used STRING database to connect 126 potential therapeutic targets and obtained a PPI network consisting of 86 nodes and 502 edges (Figure 12B) to clarify the potential mechanism of SH combined with PNC on MRSA infected wounds. Then the target protein interaction data were imported into Cytoscape software, and the topological parameters of the network were calculated and analyzed by using the plug-in CytoNCA. The key nodes in the network are screened according to Betweenness (BC), Closeness (CC), Degree (DC), Eigenvector (EC), Local Average Connectivity-based method (LAC), Network (NC) as shown in Figure 12C.

#### 3.9.3 Functional enrichment analyses and compound-target-pathways (CTP) network

DAVID database was used to conduct GO enrichment analysis and KEGG pathway enrichment analysis on the core targets to study the biological mechanism, and the biological process, cellular component and molecular function were elaborated.

**FIGURE 13:**
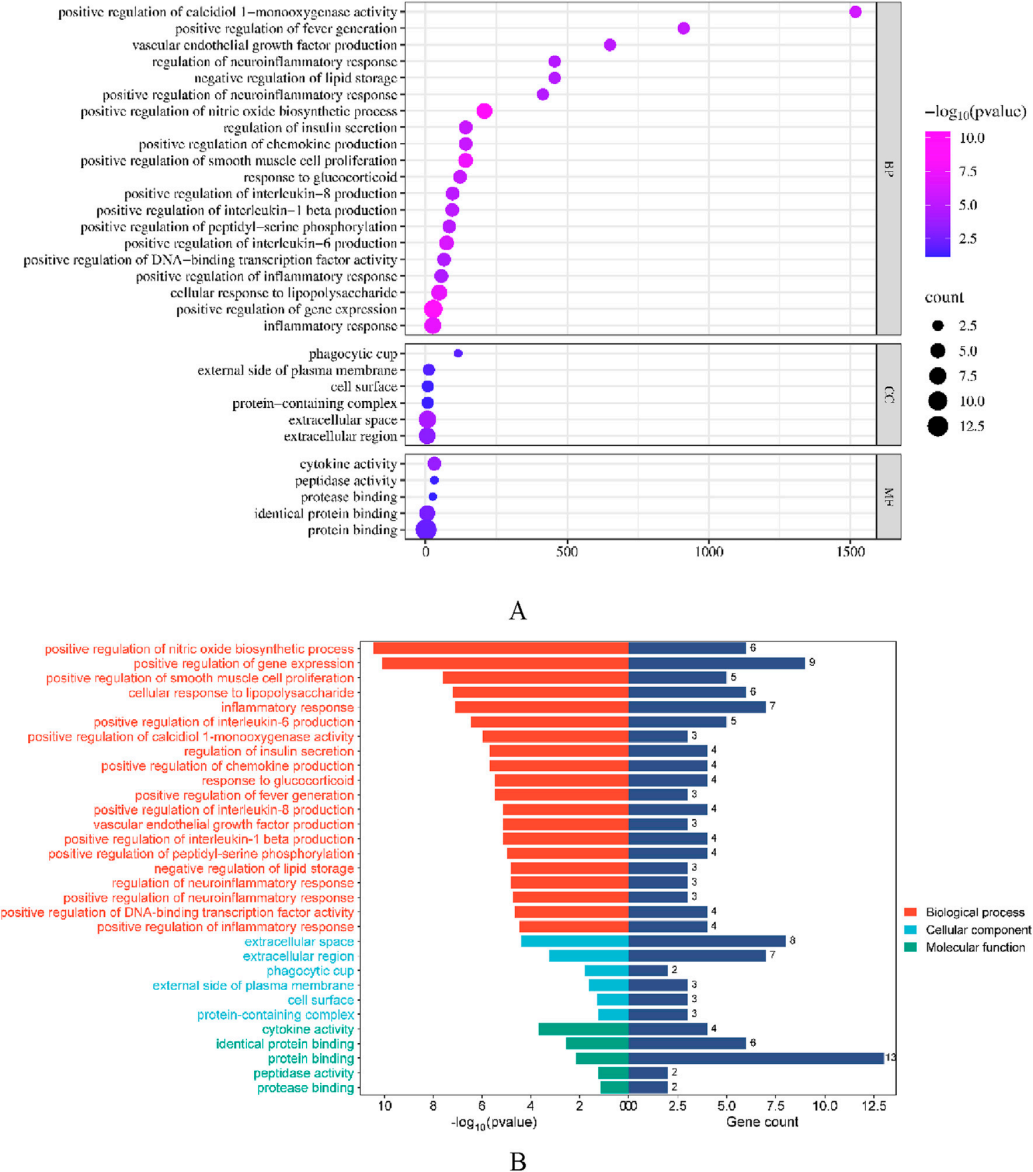
GO enrichment analysis bar chart of compounds (**(A)** is GO bubble Chart, **(B)** is bilateral bar chart).

As shown in Figure 13, the biological processes of the top five are positive regulation of nitric oxide biosynthetic process, positive regulation of gene expression, positive regulation of smooth muscle cell proliferation, cellular response to lipopolysaccharide and inflammatory response, while cellular components are extracellular space, extracellular region, phagocytic cup, external side of plasma membrane and cell surface. The mechanisms of molecular function are cytokine activity, identical protein binding, protein binding, peptidase activity, protease binding.

**FIGURE 14:**
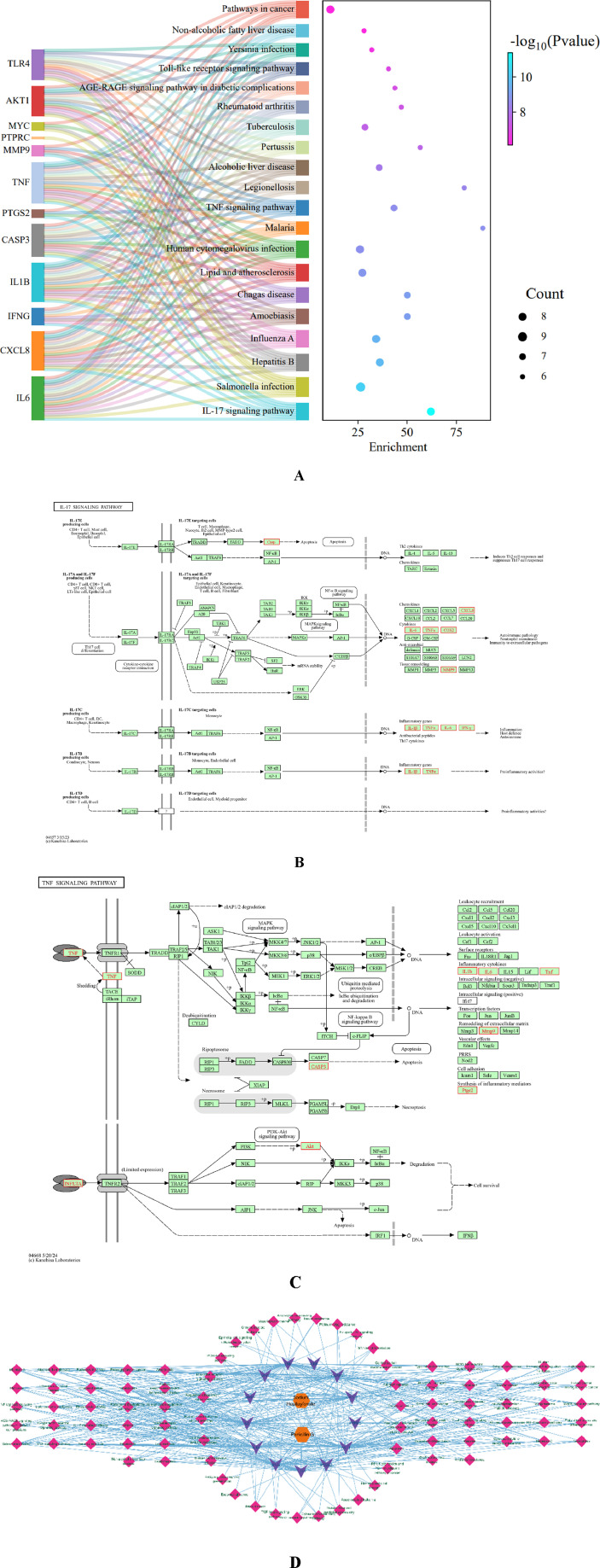
KEGG pathway Analysis Sankey + dot plot of SH + PNC **(A)** (The X-axis is the enrichment, the Y-axis is the pathway name, and the color represents the size of the p value. The smaller the p value, the redder the color, and the more significant the enrichment. The size of the sphere represents the number of targets). Molecular pathways and specific targets of action. **(B)** is IL-17 signaling pathway. **(C)** is TNF signaling pathway. **(B, C)** shows that there are common targets in both pathways. Compound-target-molecular pathways (**(D)**, Orange nodes are SH and PNC, purple nodes are targets, and pink nodes are molecular pathway).

The target related pathways were obtained by KEGG enrichment analysis. Meanwhile, we selected the top 20 representative pathways for visualization analysis based on gene count, enrichment and P-value (Figure 14A). The analysis results showed that IL-17 signaling pathway play an important role through cell proliferation and differentiation, apoptosis, regulation of immune inflammation, cytokine synthesis/inhibition. At the same time, the targets in the IL-7 pathway are the same as those in other pathways, and can act on multiple molecular pathways through the same target (Figures 14B, C).

To comprehensively elucidate the mechanism of the therapeutic effect, we constructed compound-target-molecular pathways through Cytoscape software, as shown in the Figure 14D. The CTP network of core targets has 174 nodes and 775 edges, and 155 related molecular pathways. CTP network intuitively shows the possible mechanism of treating nerve injury through the interaction between targets and different targets acting on the same pathway. By analyzing the above targets, we found that some of them are related to regulating mechanisms such as cell apoptosis, differentiation, and immune inflammation.

#### 3.9.4 Molecular docking

**FIGURE 15:**
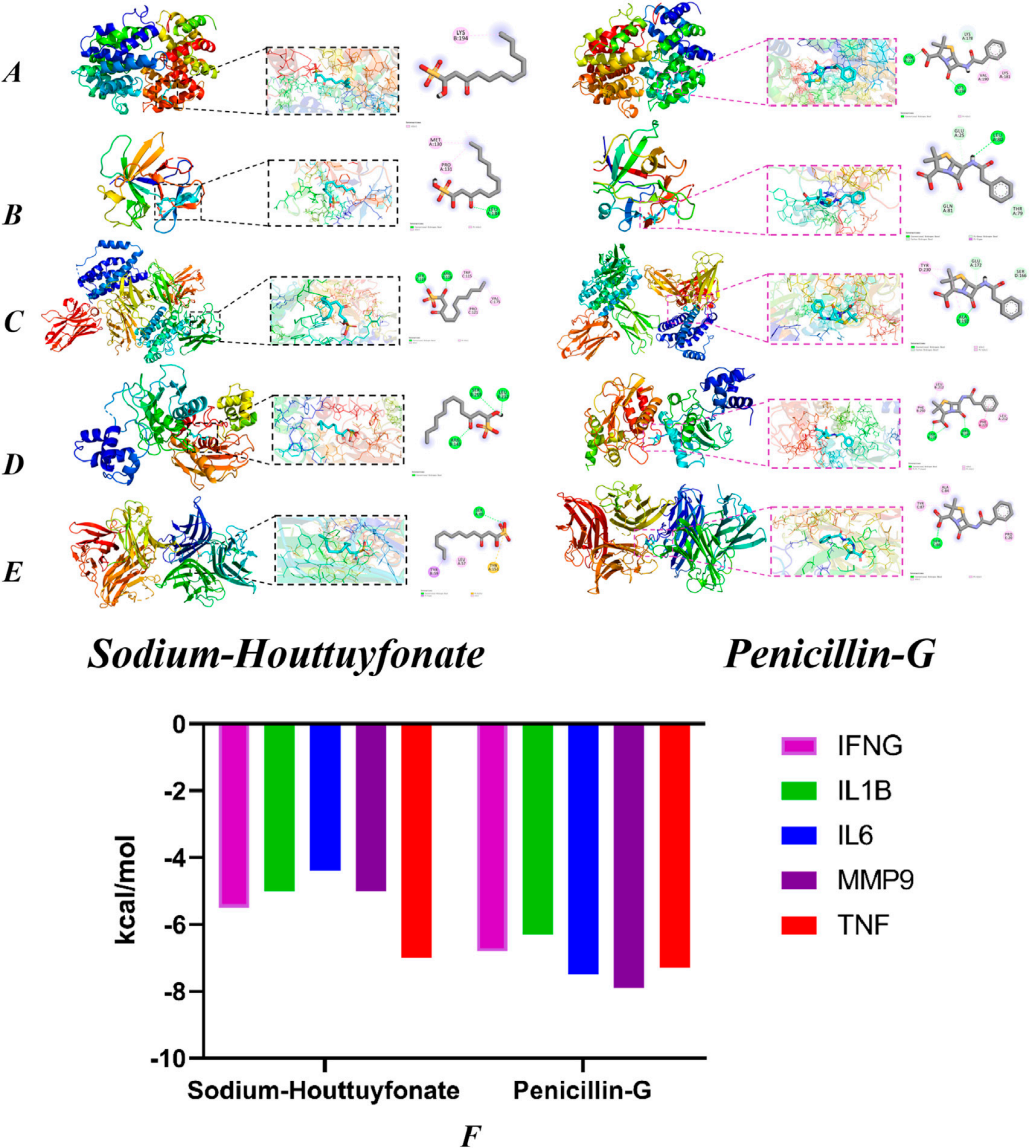
Molecular docking models of OA with possible core anti-obesity targets. IFNG **(A, B)** (IL1B), **(C)** (IL6), **(D)** (MMP9) and **(E)** (TNF). The Binding affinity of SH and PNC with various targets **(F)**.

By using CytoNCA software to reprocess the targets in the IL-17 signaling pathway, we obtained the targets IL1B, IL6, MMP9, IFNG, and TNF. Retrieve the molecular structure of the corresponding protein from the PDB database (https://www.rcsb.org/) (Konc and Janežič, 2022), and import it into AutoDock Vina to calculate the optimal binding site and binding energy (Figures 15A–E). The lower the binding energy, the higher the affinity. The binding energies of SH with IFNG, IL1B, IL6, MMP9 and TNF are −5.5, −5, −4.4, −5 and −7 kcal/mol, respectively. The PNCs are −6.8, −6.3, −7.5, −7.9 and −7.3 kcal/mol, respectively (Figure 15F).

## 4 Discussion

*Staphylococcus aureus* (S.aureus) is one of the most common opportunistic pathogens in humans, which can cause skin and soft tissue infection, intravascular infection, pneumonia, infectious arthritis, endocarditis, osteomyelitis, sepsis and other diseases (Ahmad-Mansour et al., 2021). Due to the emergence of drug-resistant strains, especially the widespread presence of MRSA, S. aureus is one of the main pathogens causing high incidence and mortality rates worldwide (Lee et al., 2018). According to the China Antimicrobial Surveillance Network report (CHINET), the isolation rate of S. aureus in China in 2020–2021 ranked first among Gram-positive bacteria, and the detection rate of MRSA was around 30%. It is one of the main infectious pathogenic microorganisms in Chinese hospitals (Yun et al., 2023).

MRSA is characterized by rapid transmission, strong pathogenicity, high mortality, and resistance to various antibiotics such as β - lactams, tetracyclines, aminoglycosides, macrolides, etc (Lakhundi and Zhang, 2018; Turner et al., 2019). It is widely believed in clinical practice that vancomycin is an effective treatment for MRSA, but it is concerning that intermediate vancomycin resistant *Staphylococcus aureus* (VISA) and vancomycin resistant *Staphylococcus aureus* (VRSA) have emerged (Hernández-Aristizábal and Ocampo-Ibáñez, 2021; Appelbaum, 2006). Therefore, it is necessary to further study the mechanism of MRSA resistance and develop new treatment plans.

Traditional Chinese medicine has a wide range of sources and multiple targets, making it difficult for bacteria to develop resistance to it. It can be used as a treatment option for drug-resistant bacteria. At present, there are many herbal extracts that can inhibit the production of bacterial biofilm or inhibit the active efflux system to reverse the antibiotic resistance of MRSA (Liu et al., 2022; Li et al., 2022). Houttuynia cordata, belongs to the family of the genus Houttuynia cordata, has the functions of clearing heat and detoxifying, eliminating carbuncles and discharging pus, diuretic and relieving stranguria, and is used for lung carbuncle, phlegm-heat asthma, hot dysentery, hot stranguria, carbuncle and sore poison. Studies shows that its extract Sodium Houttuyfonate has anti-inflammatory and antibacterial effects. At present, studies have shown that Sodium Houttuyfonate has inhibitory effects on a variety of bacteria, such as *Pseudomonas aeruginosa*, *Candida* albicans, *Acinetobacter* baumannii (Ma et al., 2021; Zhou et al., 2022b).

This article tested the antibacterial efficacy of SH *in vitro* using the micro-broth dilution method, and the results showed that SH had a certain inhibitory effect on MRSA *in vitro*. Penicillin G is one of the most commonly used antibiotics in clinical practice, with the characteristics of economy and low toxicity. Experiments have shown that the combination of SH and PNC can increase their bacterial inhibition rate, decrease the MIC of PNC, and significantly enhance the antibacterial effect of PNC.

In the MRSA-infected wound model of SD rats, it was found that the SH + PNC group could significantly reduce the bacterial count in the MRSA wound and promote the healing of the MRSA-infected wound compared with other intervention measures. The combined group can effectively reduce the content of some inflammatory factors in the blood. Pathological examination results showed that the number of neutrophils in the combined group decreased significantly and more collagen fibers were produced, suggesting that SH + PNC can regulate inflammation and promote the secretion of extracellular matrix, thereby generating granulation tissue to accelerate wound healing.

The results of network pharmacology suggest that the mechanism of SH + PNC in treating MRSA-infected wounds is complex, with the IL-17 signaling pathway believed to play an important role. The core targets of this pathway include IL1B, IL6, MMP9, IFNG, and TNF. MMP9 belongs to the matrix metalloprotein family, and its main function is to degrade and reshape the dynamic balance of extracellular matrix. The main components of extracellular matrix and vascular basement membrane are collagen and laminin (Hallmann et al., 2020; Kumar et al., 2022). As an important inflammatory cytokine, TNF-α participates in the inflammatory regulation process, which can indirectly or directly activate the expression of target genes such as NF-κB, IL-6 and IL-1β, aggravate microvascular injury and induce the production of VEGFA, and trigger more severe inflammatory reactions (Tian et al., 2024). IFNG plays a key role in driving innate and acquired defense against infectious pathogens, and Gram-positive bacteria can stimulate the expression of IFNγ, TNF-α, and IL-1β more strongly than Gram-negative bacteria (Leng et al., 2016). IFNG overexpression can also activate macrophages, neutrophils, endothelial cells, platelets, as well as the complement and coagulation systems, thereby inducing the release of bioactive substances such as tumor necrosis factor-α, histamine, serotonin, prostaglandins, and kinins (Higgs et al., 2017). Interleukin 6 (IL-6) is a pleiotropic cytokine that plays a role in immunity, tissue regeneration, and metabolism. The rapid production of IL-6 contributes to host defense during infection and tissue injury, but excessive synthesis of IL-6 and dysregulation of IL-6 receptor signaling are associated with disease pathology. Excessive IL-6 production can induce VEGF to directly or indirectly act on blood vessels, leading to increased angiogenesis activity and vascular permeability. At the same time, an increase in the Th17/Treg ratio can lead to the disruption of immune tolerance (Kang et al., 2019). As a proprotein, IL1B is produced by activated macrophages and participates in signaling pathways mainly including the NF-κB signaling pathway and the MAPK signaling pathway. It binds to its receptor IL-1RI to initiate downstream signaling, leading to the expression of inflammatory factors and acute-phase proteins (Rong et al., 2020). Molecular docking experiments showed that SH and PNC could participate in inflammatory immune regulation, apoptosis, cytokine secretion, and other processes by binding to the above targets, thereby achieving the therapeutic effect of promoting wound healing.

Although it was observed in this experiment that sodium houttuyfonate combined with penicillin G can effectively inhibit the growth of MRSA in rat models and promote the healing of MRSA-infected wounds in rats, the mechanism by which sodium houttuyfonate reverses MRSA resistance to penicillin G is still unclear. At the same time, follow-up experiments need to supplement single-cell experiments to verify the actual molecular expression and support the analysis results.

## 5 Conclusion

This study demonstrates that sodium houttuyfonate has an inhibitory effect on the growth of MRSA *in vitro* and can increase the antibacterial effect of penicillin G. It inhibits the growth of bacteria in rat MRSA infected wounds, reduces neutrophil infiltration, promotes local collagen fiber production, reduces the expression of inflammatory factors IL-6 and TNF-α, regulates local inflammatory response, and thus promotes wound healing. The IL-17 signaling pathway may play a key role in wound healing, with core targets including IL1B, IL6, MMP9, IFNG, and TNF, involving inflammatory immune regulation, apoptosis, cytokine secretion, and so on. This study provides a theoretical basis for the clinical treatment of MRSA infection with SH and subsequent related research.

## Data Availability

The original contributions presented in the study are included in the article/supplementary material, further inquiries can be directed to the corresponding authors.
